# Incidence of Post-Procedural Conduction Disturbances and Rates of Permanent Pacemaker Implantation in Older and Newer Generations of Transcatheter Aortic Heart Valves

**DOI:** 10.3390/medsci13040296

**Published:** 2025-11-30

**Authors:** Mostafa Salem, Philipp Laing, Insa Kühling-thees, Wiebke Kasper, Jakob Voran, Hatim Seoudy, Rafael Rangel, Johanne Frank, Derk Frank, Mohammed Saad

**Affiliations:** 1 Klinik für Innere Medizin III, Schwerpunkte Kardiologie, Angiologie und Intensivmedizin, Universitätsklinikum Schleswig-Holstein, 24105 Kiel, Germany; 2 Department of Internal Medicine III, Cardiology and Angiology, University Hospital Schleswig-Holstein Kiel Germany, Arnold-Heller-Str. 3, Haus K3, 24105 Kiel, Germany; 3 German Center for Cardiovascular Research (DZHK), Partner Site Hamburg/Lübeck/Kiel, 24105 Kiel, Germany

**Keywords:** aortic valve, transcatheter aortic valve replacement, new generations of aortic valve prostheses, pacemaker implantation

## Abstract

**Objective:** This analysis compares new (G2) versus old (G1) generations of transcatheter heart valves (THVs) in transcatheter aortic valve replacement (TAVR) procedures, focusing on key outcomes: post-procedural conduction disturbance (CD) and permanent pacemaker implantation (PPMI). We aim to determine whether G2 valves reduce these specific complications and thereby improve patient outcomes compared with G1. **Methods:** From February 2015 to September 2022, 1468 patients underwent TAVR at the university clinic in Kiel. After applying exclusion criteria, a final cohort of 1182 patients were analysed. Among these, 782 patients underwent TAVR with G1, whereas 400 underwent TAVR with G2. The primary study endpoints were the occurrence of new CD and PPMI within 30 days post-procedure. The secondary endpoints included diverse post-TAVR events as defined by the safety criteria of Valve Academic Research Consortium 3 (VARC III). A statistical analysis compared outcomes between the G1 and G2 groups. **Results:** Out of 1182 patients, 12.1% required PPMI within 30 days. Rates showed no statistical difference between G2 and G1 for PPMI (10.3% vs. 13.0%, IPTW-weighted *p* = 0.31) or CD (15.3% vs. 21.48%, IPTW-weighted *p* = 0.08). Among G2, the Sapien 3 Ultra valve had the lowest PPMI rate (4.8%). Overall, G2 and G1 had similar post-procedural and 30-day mortality rates. **Conclusion:** G2 valves may reduce post-procedure CD, but the difference is not statistically significant. Differences between specific valve types—such as the Sapien 3 Ultra’s lower rates—are notable, but overall, PPMI and safety profiles remain similar between G1 and G2. Patient and procedural factors still play a significant role. Careful valve and patient selection is essential, and ongoing research will guide further improvements.

## 1. Introduction

Transcatheter aortic valve replacement (TAVR) has become a widely recognised approach for managing severe aortic stenosis (AS) across different patient risk levels. Initially reserved for those at high risk or deemed inoperable, TAVR has now extended its reach to include intermediate [1,2] and elderly lower-risk patients [3,4,5]. Despite advancements in technique and device technology, complications such as new conduction disturbances (CDs) requiring new permanent pacemaker implantation (PPMI) remain significant concerns. These complications have been reported at rates ranging from 3.4% to 25.9% [4,6]. They are associated with increased hospital stays and costs, accounting for about 25% of TAVR procedure expenses [7]. They also correlate with a poorer long-term prognosis [8]. Key predictors of conduction issues post-TAVR include pre-existing right bundle branch block (RBBB), self-expanding prostheses (SE), pre-and post-balloon dilation, and a deeper valve implantation [4,9].

Technological strides continue to improve TAVR’s technical success and reduce the rate of adverse events. Significant reductions in paravalvular leak (PVL) have been achieved by integrating an external sealing “skirt” into the SAPIEN-3 (S3) valve with a balloon-expandable mechanism (BE) [10]. Compared to the earlier S3, the updated Sapien 3 Ultra (S3U) is better suited for complex vascular anatomy due to its enhanced delivery force, which prevents overinflation of the deployment balloon and ensures optimal valve leaflet coaptation [11].

Modifications to the SE transcatheter heart valve (THV) in the Evolut R (ER) system have shortened and reshaped its outflow, aiming to better align with the native sinus and thereby reduce load on the conduction system, potentially decreasing the need for PPMI. The newer generation, named Evolut PRO (EP) valve, is built on this platform by incorporating an external pericardial tissue wrap at the inflow to minimise PVL [12].

Moreover, Abbot’s latest valve generation, the Navitor Valve, features innovative enhancements, such as a polyethene sealing cuff (Naviseal, Abbott Inc., Chicago, IL, USA) designed to reduce PVL, and structural refinements to the titanium–nickel alloy frame to uniformise radial force distribution across various valve sizes [13].

Despite technological advances in newer-generation TAVR prostheses (G2), comparative data on the incidence of PPMI between older-generation (G1) and G2 devices remain inconclusive [10,14]. Therefore, this study aims to evaluate the impact of THV generation (G1 vs. G2) on 30-day rates of new CD and PPMI, and to compare 30-day and 1-year mortality, as well as technical success according to the VARC-III criteria, between the two groups.

## 2. Methods

### 2.1. Study Population and Design

A retrospective, observational study was conducted at a single institution. The data, including demographic details, clinical characteristics, in-hospital treatments, and follow-up information, were collected through the electronic medical archive system, which features platforms such as Orbis^®^, Enaio^®^, and Meona^®^ alongside patient files.

The study focused on consecutive patients who received TAVR for severe AS. The TAVR procedures utilised both G1 devices, such as the Evolut R (Edwards Lifesciences, Irvine, CA, USA), Sapien 3 (Edwards Lifesciences, Irvine, CA, USA), Accurate Neo (Boston Scientific, Marlborough, MA, USA) and Centera (Centera Photonics Inc., Hsinchu City, Taiwan), and G2 devices, including the Evolut Pro (MEDTRONIC INC., Minneapolis, MN, USA), Sapien 3 Ultra (Edwards Lifesciences, Irvine, CA, USA), Accurate Neo 2 and Navitor (The Navigator Company, Setubal, Portugal) (Table 1). These procedures were conducted at the University Heart Hospital Kiel from February 2015 to September 2022. A multidisciplinary heart team consistently guided the treatment decision-making process, selecting the most suitable strategy—from surgical aortic valve replacement and TAVR to medical management—based on prevailing clinical guidelines.

### 2.2. Inclusive and Exclusive Criteria

The study focused on patients selected for TAVR. It employed specific exclusion criteria, ruling out patients under 18 years of age, those unable to provide informed consent, those requiring non-transfemoral approaches, those with bicuspid aortic valves, or those who had previously received pacemaker implantations. Initially, 1468 patients were considered; after applying the exclusion criteria, 1182 remained eligible for inclusion in the study.

The study protocol was approved by the local ethics committee of the University Hospital Schleswig-Holstein, Campus Kiel (Ethics Committee of the Medical Faculty, Christian-Albrechts-University of Kiel; approval no. D 506/22) and conducted in accordance with the Declaration of Helsinki. Written informed consent was obtained from all participants (or their legal representatives).

### 2.3. Transcatheter Aortic Valve Replacement Procedure (TAVR) and Valve Selection [15]

All procedural aspects, including valve selection, anaesthesia type, and access site, were selected at the operator’s discretion, guided by the patient’s clinical profile, anatomical considerations, and angiographic findings. Prostheses were sized according to manufacturer guidelines, which included measuring annulus and left ventricular outflow tract (LVOT) dimensions and assessing the extent of calcification in these areas.

The THV was positioned and implanted using either the standard three-cusp coplanar projection or the advanced cusp overlap technique, selected based on pre-procedural CT scan evaluations and fine-tuned during angiography just before the procedure. Proper alignment and deployment of the THV began with the delivery catheter crossing the aortic valve, adjusting its position at the annulus or slightly below (not exceeding 1 mm). Cranial or caudal angulation adjustments were made to minimise THV parallax. The positioning was meticulously controlled by monitoring the contact between the THV and the non-coronary cusp (NCC) until it reached the left coronary cusp (LCC), with careful tension management or slight retraction of the delivery system to avoid encroachment into the LVOT.

After initial positioning, any necessary adjustments or repositioning of the THV were made at the operator’s discretion to ensure optimal placement about 2 to 4 mm below the annulus. Occasionally, rapid pacing at 120–140 bpm was used during valve deployment at the operator’s discretion.

Each patient underwent a 12-lead electrocardiogram before the procedure, which was repeated 24 h after the procedure, at discharge, and 30 days after TAVR. Consideration for PPMI was given to patients who exhibited persistent complete atrioventricular block (AVB), those with existing conduction abnormalities such as RBBB or first-degree AVB who developed high-grade AVB during or after THV deployment without spontaneous recovery within 24 h, or those without prior conduction issues who developed high-grade AVB post-procedure after 24 h.

### 2.4. Study Objectives

The primary objective of this study was to analyse the occurrence and rate of new CD and PPMI within 30 days post-procedure between the two groups, G1 and G2. Secondary objectives focused on assessing several safety metrics defined by the Valve Academic Research Consortium 3 (VARC-3), including post-TAVR incidents such as 30-day all-cause mortality, myocardial infarction, stroke, vascular complications, and major and minor bleeding events.

### 2.5. Statistical Methods

All continuous variables were tested for normality using the Kolmogorov–Smirnov test. All continuous data are summarised as mean ± standard deviation, and categorical variables as counts and percentages. For the descriptive comparison of peri- and post-procedural characteristics between patients treated with G1 and G2, normally distributed continuous variables were compared using the independent sample *t*-test, and non-normally distributed variables were compared using the Wilcoxon rank-sum test. Categorical variables were compared using Pearson’s chi-square test or Fisher’s exact test, as appropriate. Baseline characteristics are presented descriptively without formal hypothesis testing; covariate balance between G1 and G2 was assessed using absolute standardised mean differences (ASMDs).

To account for baseline imbalances and potential confounding in the comparison of outcomes between G1 and G2, we applied inverse probability of treatment weighting (IPTW) [16]. A propensity score for treatment with G2 was estimated for each patient using a logistic regression model with THV generation (G2 vs. G1) as the dependent variable and the following covariates as independent variables: age, sex, diabetes, dyslipidaemia, hypertension, atrial fibrillation/flutter, prior coronary artery disease, prior cardiac surgery, chronic obstructive pulmonary disease, peripheral arterial disease, cerebrovascular disease, baseline left ventricular ejection fraction, aortic valve mean pressure gradient, systolic pulmonary artery pressure, STS score and EuroSCORE II. IPTW was then calculated as 1/PS for G2 patients and 1/(1–PS) for G1 patients and applied as case weights in subsequent models. For prespecified binary endpoints (new CD, LBBB, new PPMI, post-procedural MI, stroke and vascular access site complications), IPTW-adjusted odds ratios (OR) with 95% confidence intervals (CI) were obtained from weighted logistic regression models with THV generation as the sole predictor. Survival up to 1 month and 1 year was analysed using the Kaplan–Meier method, and survival distributions between G1 and G2 were compared with the log-rank test. All tests were two-sided, and *p*-values < 0.05 were considered statistically significant. Statistical analyses and graphs were performed using Analyse-it Version 6.15 (Analyse-it Software Ltd., Leeds, UK) integrated with Microsoft Excel and IBM SPSS Statistics, version 31 (IBM Corp., Armonk, NY, USA).

## 3. Results

### 3.1. Baseline Characteristics of the Study Population (Table 2)

Between February 2015 and September 2022, our centre performed 1486 TAVR procedures. Of these, 1182 patients were selected for the current analysis after applying our exclusive criteria. Among the included patients, 782 were treated with the G1 and 400 with the G2 THV.

Table 2 summarises the baseline characteristics of the study population. The average age of the participants was 82 years (±6). Females constituted 49.7% of the cohort (588 patients). The distribution of cardiovascular risk factors and relevant past medical histories was similar between the two groups.

Most patients were electively admitted with an intermediate surgical risk and were evenly distributed across the two generations, as indicated by mean EuroSCOREs II of 17.83 (±13.09) for G1 and 17.14 (±12.42) for G2. The Society of Thoracic Surgeons score was 4.46 (±3.47) for G1 and 3.89 (±2.50) for G2.

When comparing the two groups, the prevalence of hypertension was similar (89.9% in G1 vs. 89.2% in G2). However, a higher percentage of G1 patients were in New York Heart Association functional class III or IV (73.4% vs. 70.5%). The baseline left ventricular ejection fraction was nearly identical (52.6% ± 10.62 for G1 vs. 54.13% ± 9.34 for G2).

The primary indication for TAVR in all cases was severe AS. The mean aortic valve gradient was 40.12 mmHg (±15.27) for G1 and 42.52 mmHg (±15.7) for G2, and the aortic opening area was 0.75 (±0.17) for G1 compared to 0.71 (±0.18) for G2.

No formal hypothesis testing was performed for baseline comparisons. Overall, the variables between the two groups remained broadly similar.

**Table 2:** Baseline characteristics.

Characteristic	G1, *n* = 782 ^1^	G2, *n* = 400 ^1^
Age (years)	81.74 (±5.58)	82.04 (±5.92)
female	378/782 (48.3%)	210/400 (52.5%)
Height (cm)	169.55 (±9.46)	168.63 (±8.56)
Weight (kg)	78.15 (±16.30)	76.75 (±16.10)
BMI [kg/m^2^]	27.10 (±4.95)	28.69 (±8.69)
Diabetes	256/782 (32.7%)	105/400 (26.3%)
Dyslipidaemia	436/782 (55.8%)	253/400 (63.3%)
Hypertension	703/782 (89.9%)	357/400 (89.3%)
Atrial fibrillation or flutter	338/782 (43.2%)	123/400 (30.8%)
CAD	519/782 (66.4%)	232/400 (58.0%)
Previous cardiac surgery	126/782 (16.1%)	41/400 (10.3%)
COPD	97/782 (12.4%)	30/400 (7.5%)
PAD	61/782 (7.8%)	26/400 (6.5%)
Cerebrovascular disease	130/782 (16.6%)	48/400 (12.0%)
Pre dialysis	9/782 (1.2%)	3/400 (0.8%)
NYHA class		
I	34/782 (4.4%)	33/400 (8.3%)
II	174/782 (22.3%)	85/400 (21.3%)
III	490/782 (62.7%)	239/400 (59.8%)
IV	84/782 (10.7%)	43/400 (10.8%)
STS Score [%]	4.46 (±3.47)	3.89 (±2.50)
Log EuroScore	17.83 (±13.09)	17.14 (±12.42)
EuroScore II	4.46 (±3.47)	3.89 (±2.50)
Ejection fraction (%)	52.60 (±10.62)	54.13 (±9.34)
Ejection fraction category		
I	524/782 (67.0%)	295/400 (73.8%)
II	134/782 (17.1%)	68/400 (17.0%)
III	72/782 (9.2%)	21/400 (5.3%)
IV	52/782 (6.7%)	16/400 (4.0%)
AV-MPG (mmHg)	40.12 (±15.27)	42.52 (±15.77)
sPAP (mmHg)	44.19 (±13.89)	42.50 (±13.37)
AVA [cm^2^]	0.75 (±0.17)	0.71 (±0.18)

^1^ Mean (SD); n/N (%). Values are unweighted. G1: old generation, G2: new generation, BMI: body-mass index, CAD: coronary artery disease, COPD: chronic obstructive pulmonary disease, PAD: peripheral arterial occlusive disease, NYHA: New York Heart Association classification, STS Score: Society of Thoracic Surgeons score, AV-MPG: aortic valve mean pressure gradient, sPAP: systolic pulmonary artery pressure, AVA: aortic valve area.

### 3.2. Peri- and Post-Procedural Data and Outcomes (Table 3; Unweighted, Unadjusted and Table 4; IPTW-Adjusted)

The study compared peri- and post-procedural outcomes between the two generations. In the crude, unweighted analysis, G2 was associated with a significantly lower incidence of new CD compared with G1 (15.3% vs. 21.5%; *p* = 0.013). After IPTW adjustment, this difference was attenuated and no longer statistically significant, although the point estimates still suggested a numerically lower risk with G2 (OR 0.63; 95% CI 0.37–1.05; *p* = 0.08).

Further subcategorisation of CD showed that only LBBB differed significantly between the two generations. In both unweighted and IPTW-adjusted analyses, G2 had a lower incidence of LBBB than G1 (4.0% vs. 9.5%; unweighted *p* = 0.001; IPTW-adjusted OR 0.36, 95% CI 0.14–0.91; *p* = 0.03), whereas all other CD subtypes (AV block I–III, SSS, RBBB, SVT) showed no statistically significant differences, either before or after IPTW. These findings suggest that technological refinements in G2 THV may reduce cardiac conduction injury, particularly new LBBB, the most common post-TAVR CD, and are consistent with a more favourable overall conduction safety profile.

However, the overall rate of 30-day post-procedural PPMI did not differ significantly between G1 and G2 in either unweighted or IPTW-adjusted analyses, and the crude proportions were also nearly similar (13.04% vs. 10.25%; unweighted *p* = 0.194; IPTW-adjusted OR 0.72, 95% CI 0.39–1.35; *p* = 0.31). This highlights the complexity of factors influencing pacemaker requirements after TAVR, which extend beyond generational changes in THV technology.

Additionally, significant differences were observed in contrast agent dose and procedure duration. The G2 valves required significantly fewer contrast agents than the G1 valves, with an average usage of 79.05 mL (±33.41) for G2 versus 88.32 mL (±30.71) for G1, resulting in a significant reduction (unweighted *p*-value < 0.01; IPTW-weighted *p* < 0.01). Moreover, the average procedure time for G2 was approximately 10 min shorter than for G1, with mean durations of 44.12 min (±17.85) for G2 and 53.08 min (±21.43) for G1 (unweighted *p*-value < 0.01; IPTW-weighted *p* < 0.01).

The analysis of post-procedural outcomes according to the secondary endpoints and VARC-3 criteria showed no statistically significant differences between G1 and G2 THV in either unweighted or IPTW-weighted analyses for most major safety endpoints, although some numerical trends were observed. Post-procedural myocardial infarction was slightly less frequent in G2 than G1 (0.25% vs. 0.38%; unweighted *p* > 0.9; IPTW-adjusted OR 0.53, 95% CI 0.02–19.16; *p* = 0.73). Stroke occurred more often in G2 than G1 (2.25% vs. 1.53%; unweighted *p* = 0.364; IPTW-adjusted OR 1.62, 95% CI 0.42–6.14; *p* = 0.48), with similar patterns for ischaemic and haemorrhagic subtypes. Disabling stroke was numerically more frequent in G2 than G1, but without statistical significance (1.75% vs. 0.51%; unweighted *p* = 0.052; IPTW-adjusted OR 3.79, 95% CI 0.65–22.07; *p* = 0.14). Vascular access site complications were also slightly more common in G2 than G1 (4.50% vs. 3.58%; unweighted *p* = 0.81; IPTW-adjusted OR 1.18, 95% CI 0.46–3.02; *p* = 0.73), a pattern that was similar for both major and minor events. Overall, these findings suggest that post-procedural safety, as assessed by VARC-3, remains broadly comparable between G1 and G2 THV. The detailed results are summarised in Table 3 and Table 4.

**Table 3:** Peri- and post-procedural data and outcomes (unweighted, unadjusted).

Characteristic	G1, *n* = 782 ^1^	G2, *n* = 400 ^1^	Unweighted *p*-Value ^2^
New conduction disturbance	168/782 (21.5%)	61/400 (15.3%)	0.01
Type conduction disturbance			0.24
AVB I	12/782 (1.5%)	6/400 (1.5%)	>0.90
AVB II	5/782 (0.6%)	2/400 (0.5%)	>0.90
AVB III	78/782 (10.0%)	32/400 (8.0%)	0.32
SSS	17/782 (2.2%)	11/400 (2.8%)	0.55
LBBB	74/782 (9.5%)	16/400 (4.0%)	0.001
RBBB	4/782 (0.5%)	1/400 (0.3%)	0.67
SVT	7/782 (0.9%)	2/400 (0.5%)	0.73
Valve in valve	22/782 (2.8%)	13/400 (3.2%)	0.81
New pacemaker implantation	102/782 (13.0%)	41/400 (10.3%)	0.19
Type of pacemaker			<0.001
VVI-PM	16/782 (2.1%)	5/400 (1.3%)	
DDD-PM	67/782 (8.6%)	33/400 (8.3%)	
CRT-P	15/782 (1.9%)	3/400 (0.8%)	
Valve type			<0.001
Evolut^TM^ R	465/782 (59.5%)	0/400 (0.0%)	
Evolut^TM^ PRO	0/782 (0.0%)	146/400 (36.5%)	
SAPIEN 3	314/782 (40.2%)	0/400 (0.0%)	
SAPIEN 3 Ultra	0/782 (0.0%)	207/400 (51.8%)	
ACURATE neo	3/782 (0.4%)	0/400 (0.0%)	
ACURATE neo2	0/782 (0.0%)	5/400 (1.3%)	
Navitor	0/782 (0.0%)	30/400 (7.5%)	
Centra	0/782 (0.0%)	12/400 (3.0%)	
Valve type (release function)			<0.001
self-expanding valves	468/782 (59.9%)	193/400 (48.3%)	
balloon-expandable valves	314/782 (40.2%)	207/400 (51.8%)	
Valve size			<0.001
20 mm	0/782 (0.0%)	3/400 (0.8%)	
23 mm	107/782 (13.7%)	65/400 (16.3%)	
25 mm	2/782 (0.3%)	9/400 (2.3%)	
26 mm	222/782 (28.4%)	182/400 (45.5%)	
27 mm	1/782 (0.1%)	11/400 (2.8%)	
29 mm	317/782 (40.5%)	114/400 (28.5%)	
34 mm	133/782 (17.0%)	16/400 (4.0%)	
Procedure Duration [min]	53.08 (±21.4)	44.12 (±17.9)	<0.001
Contrast agent used [mL]	88.32 (±30.7)	79.05 (±33.4)	<0.001
Post-procedural myocardial infarction	3/782 (0.4%)	1/400 (0.3%)	>0.90
Stroke	12/782 (1.5%)	9/400 (2.3%)	0.36
ischaemic	11/782 (1.4%)	8/400 (2.0%)	0.47
haemorrhagic	1/782 (0.1%)	1/400 (0.3%)	>0.90
undetermined	8/782 (1.0%)	2/400 (0.5%)	0.51
disabling	4/782 (0.5%)	7/400 (1.8%)	0.05
Vascular access site complications	34/782 (3.6%)	17/400 (4.5%)	0.81
major	9/782 (1.2%)	4/400 (1.0%)	>0.90
minor	25/782 (3.2%)	13/400 (3.3%)	0.75

^1^ Mean (±SD); n/N (%). Values are unweighted. ^2^ *p*-values represent unadjusted group comparisons (Wilcoxon rank-sum test; Pearson’s chi-square test; Fisher’s exact test). Inverse probability of treatment weighting (IPTW)-adjusted associations between THV generation and key outcomes are reported in Table 4. AVB: atrioventricular block, LBBB: left bundle branch block, RBBB: right bundle branch block, SSS: sick sinus syndrome, AF: atrial fibrillation, VT: ventricular tachycardia, VVI-PM: one-chamber pacemaker, DDD-PM: two-chamber pacemaker, CRT-P: cardiac resynchronisation therapy with a pacemaker, S-ICD: subcutaneous implantable cardioverter defibrillator.

**Table 4:** IPTW-adjusted association between THV generation and outcomes.

Characteristic	G1, Crude n/N (%)*^1^*	G2, Crude n/N (%) ^1^	IPTW-Adjusted OR (G2 vs. G1) ^2^	95% CI ^2^	Weighted *p*-Value ^2^
New conduction disturbance	168/782 (21.5%)	61/400 (15.3%)	0.63	0.37–1.05	0.08
Type conduction disturbance					
AVB I	12/782 (1.5%)	6/400 (1.5%)	0.53	0.067–4.22	0.55
AVB II	5/782 (0.6%)	2/400 (0.5%)	1.74	0.19–16.2	0.63
AVB III	78/782 (10.0%)	32/400 (8.0%)	0.77	0.39–1.53	0.46
SSS	17/782 (2.2%)	11/400 (2.8%)	0.62	0.12–3.13	0.56
LBBB	74/782 (9.5%)	16/400 (4.0%)	0.36	0.14–0.91	0.03
RBBB	4/782 (0.5%)	1/400 (0.3%)	0.53	0.02–19.16	0.73
SVT	7/782 (0.9%)	2/400 (0.5%)	0.61	0.05–7.84	0.70
Valve in valve	22/782 (2.8%)	13/400 (3.2%)	1.53	0.55–4.22	0.41
New pacemaker implantation	102/782 (13.0%)	41/400 (10.3%)	0.72	0.39–1.35	0.31
Post-procedural myocardial infarction	3/782 (0.4%)	1/400 (0.3%)	0.53	0.02–19.16	0.73
Procedure duration [min]	53.08 (±21.4)	44.12 (±17.9)	--	--	<0.001
Contrast agent used [mL]	88.32 (±30.7)	79.05 (±33.4)	--	--	<0.001
Stroke	12/782 (1.5%)	9/400 (2.3%)	1.62	0.42–6.14	0.48
ischaemic	11/782 (1.4%)	8/400 (2.0%)	1.56	0.38–6.39	0.53
haemorrhagic	1/782 (0.1%)	1/400 (0.3%)	2.14	0.03–135.1	0.72
undetermined	8/782 (1.0%)	2/400 (0.5%)	0.53	0.04–6.72	0.63
disabling	4/782 (0.5%)	7/400 (1.8%)	3.79	0.65–22.07	0.14
Vascular access site complications	34/782 (3.6%)	17/400 (4.5%)	1.18	0.46–3.02	0.73
major	9/782 (1.2%)	4/400 (1.0%)	1.04	0.16–6.81	0.97
minor	25/782 (2.8%)	13/400 (1.5%)	1.23	0.42–3.58	0.71

^1^ Crude values (columns 2–3) are unweighted and expressed as mean ± SD or n/N (%). ^2^ OR = odds ratio; CI = confidence interval. IPTW-adjusted ORs (columns 4–6) are obtained from weighted logistic regression models with G2 THV as the exposure. The propensity model included age, sex, diabetes, dyslipidaemia, hypertension, atrial fibrillation, prior coronary artery disease, prior cardiac surgery, COPD, PAD, cerebrovascular disease, baseline LVEF, AV mean gradient, systolic pulmonary artery pressure, STS score and EuroSCORE II. AVB: atrioventricular block, LBBB: left bundle branch block, RBBB: right bundle branch block, SSS: sick sinus syndrome, AF: atrial fibrillation, VT: ventricular tachycardia, VVI-PM: one-chamber pacemaker, DDD-PM: two-chamber pacemaker, CRT-P: cardiac resynchronisation therapy with a pacemaker, S-ICD: subcutaneous implantable cardioverter defibrillator.

**Figure 1:**
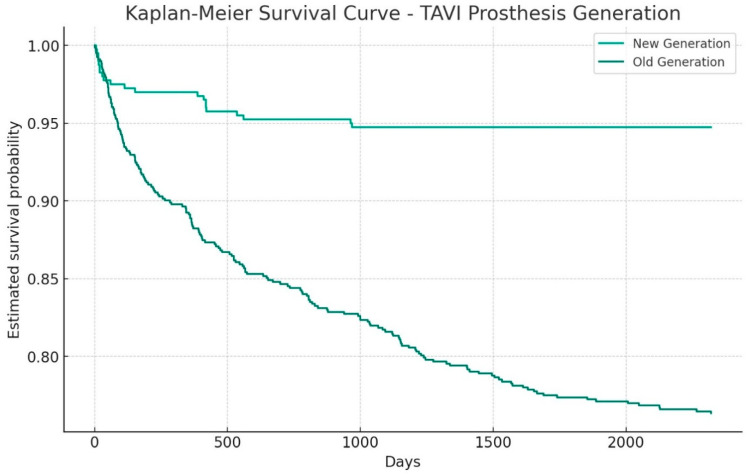
Kaplan–Meier survival analysis.

The Kaplan–Meier (Figure 1) method was used to compare survival probabilities between patients in G1 and G2. The survival probabilities at 1 month and 1 year were calculated for both groups.

At 1 month, the survival probability was 98.47% for the G1 group and 98.99% for the G2 group. At 1 year, the survival probability was 88.62% for the G1 group and 97.98% for the G2 group.

A log-rank test was performed to assess differences in survival distributions between the two groups. The test yielded a *p*-value of 0.01, indicating a statistically significant difference in survival rates between G1 and G2 over 1 year. This comprehensive analysis highlights the differences in the performance of the two generations of TAVI prostheses.

Time-to-event outcomes (30-day and 1-year mortality) were analysed using unweighted Kaplan–Meier curves and log-rank tests, because SPSS Statistics 31 does not support Cox or log-rank analyses with non-integer inverse probability of treatment weights. Given good covariate balance after IPTW and the relatively low number of events, we considered an unweighted survival analysis appropriate and present it descriptively.

### 3.3. Comparative Analysis of Valve Types

A deeper dive into the specific valve types used in G2 versus G1 revealed varying profiles in procedural outcomes. Newer models, such as the EP and S3U, have demonstrated lower CD and PPMI rates than earlier models, the ER and S3. This variance highlights the importance of valve design in influencing clinical outcomes. It may guide future device selection and procedural planning (Table 1)

The manuscript includes graphical figures that clearly show these findings. Separate figures illustrate the occurrence of new CD and the 30-day post-procedural PPMI rates for various valve models, release mechanisms, and valve generations. These visual aids are important for conveying the effects of valve selection on clinical outcomes and for presenting a brief, immediately comprehensible comparative perspective.

**Figure 2:**
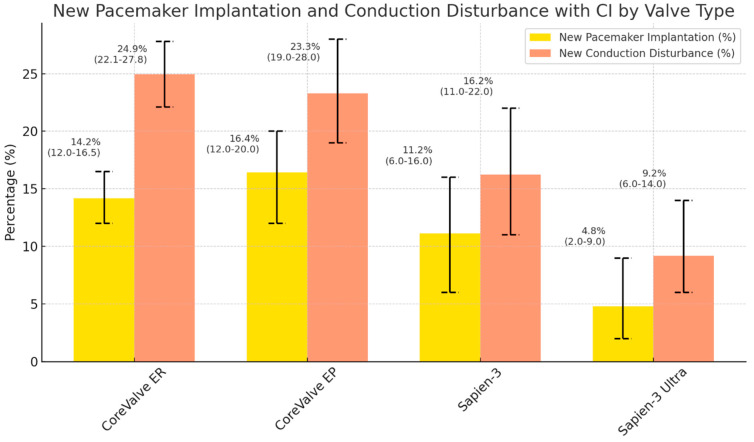
Crude incidence of new PPMI and new CD after TAVR according to the four most frequently used THV types (CoreValve ER, CoreValve EP, Sapien 3 and Sapien 3 Ultra). Bars show percentages with 95% binomial confidence intervals; yellow bars indicate PPMI and orange bars indicate CD. THV types used only rarely in our cohort (Acurate Neo/Neo2, Navitor, Centera; each *n* ≤ 30) are not displayed.

Figure 2. Percentage and 95% CI of new PPMI and CD by valve type.

In the four main THV types shown in Figure 2, crude PPMI rates ranged from 4.8% with Sapien 3 Ultra to 16.4% with CoreValve EP, whereas CD rates ranged from 9.2% (Sapien 3 Ultra) to 24.9% (CoreValve EP). Sapien 3 Ultra consistently showed the lowest incidence of both PPMI (4.8%) and CD (9.2%), while the early CoreValve platforms (ER and EP) had higher rates of conduction abnormalities (PPMI 14.2–16.4%; CD 23.3–24.9%).

**Figure 3:**
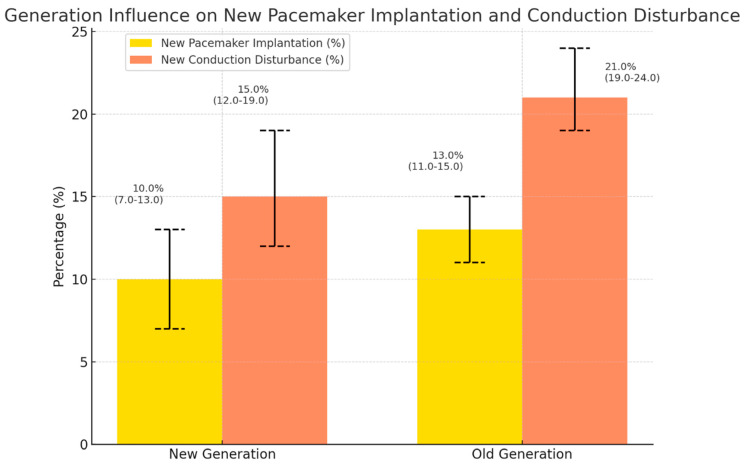
Crude incidence of new PPMI and new CD after TAVR according to THV generation. Bars show percentages with 95% confidence intervals (binomial), separately for new pacemaker implantation (yellow) and new conduction disturbance (orange).

Figure 3. Generation influence on new PPMI and CD. 

Figure 3 shows the incidence of new PPMI and new CD after TAVR according to THV generation. Bars represent percentages with 95% binomial confidence intervals. The new CD was less frequent in G2 than in G1 (15.25% vs. 21.48%), and the IPTW-weighted analysis showed a trend toward lower risk in G2 (OR 0.63, 95% CI 0.37–1.05; weighted *p* = 0.08), although this did not reach conventional statistical significance. In contrast, PPMI rates were similar between generations (10.25% vs. 13.04% for G2 and G1, respectively; weighted *p* = 0.31).

**Figure 4:**
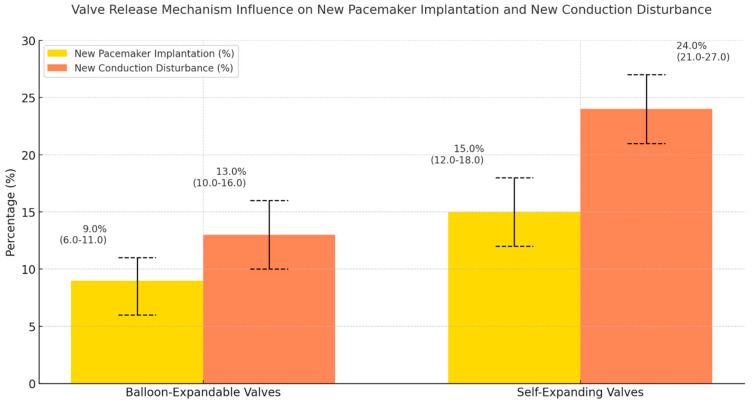
Crude incidence of new PPMI and new CD after TAVR according to THV release mechanisms. Bars show percentages with 95% confidence intervals (binomial), respectively, for new pacemaker implantation (yellow) and new conduction disturbance (orange).

Figure 4. Valve release mechanism influence on new PPMI and CD. 

This figure explores how different valve release mechanisms—specifically balloon-expandable (BE) versus self-expanding (SE) affect new PPMI and CD. BE valves are associated with significantly lower rates of both outcomes than SE valves, with 8.64% for PPMI and 13.44% for CD, compared with 14.83% and 24.05%, respectively, for SE valves. This significant difference (PPMI unweighted *p* = 0.001; CD unweighted *p* < 0.001) underscores the crucial role of valve expansion mechanisms in postoperative outcomes.

**Figure 5:**
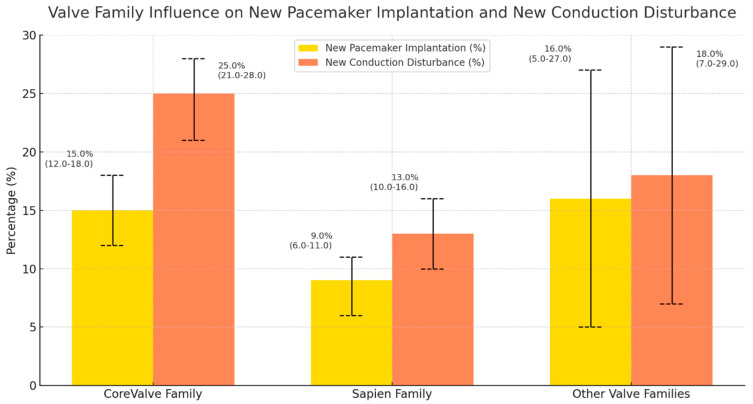
Crude incidence of new PPMI and new CD after TAVR according to THV family. Bars show percentages with 95% confidence intervals (binomial), respectively, for new pacemaker implantation (yellow) and new conduction disturbance (orange).

Figure 5. Valve family impact on new PPMI and CD. 

This figure categorises the results by valve family, explicitly comparing the CoreValve, Sapien, and other families. The Sapien family shows the lowest rates of both PPMI (8.6%) and new CD (13.4%), significantly outperforming the CoreValve family (14.7% and 24.6%, respectively) and other valve groups (16.0% and 18.0%). This suggests that Sapien valves may offer a safer profile on post-procedural cardiac electrical disturbances, with significant differences observed in PPMI (*p* = 0.06) and CD (*p* < 0.001).

## 4. Discussion

TAVI has been a significant treatment for severe symptomatic AS in patients across all surgical risk categories for more than a decade [1,3]. Despite its high success rate and improved clinical outcomes, adverse events remain a concern. The newer generations of THV were specifically developed to reduce the frequency of these complications [17,18].

An earlier randomised study comparing the early first-generation THV, specifically the Medtronic CoreValve, with the Edwards SAPIEN XT, revealed increased occurrences of PVL and valve displacement ‘popping-out’ in patients receiving the CoreValve [19]. In response, newer models like the S3 and ER were developed to enhance procedural success and safety. The S3, for instance, was specifically engineered to reduce PVL compared with earlier models by including a paravalvular sealing mechanism [20], as PVL is a recognised predictor of poor outcomes in TAVR procedures [21]. However, this modification also increases the incidence of new PPMI [22]. The added skirt, designed to minimise PVL, inadvertently increased trauma to the cardiac conduction system, thereby increasing the need for PPMI [23]. So, there has been an inverse trend between the rates of PVL (from 11.8% to 3.7%) and PPMI (from 3.8% to 8.5%), moving from the earlier Sapien XT to our G1 valve, the S3 model [24].

A recent study [25] also showed that the incidence of PPMI remains a significant concern post-TAVR, even with the development of newer-generation valves. Given that the predictors of this event are well-known, it is essential to identify patients at increased risk for PPMI proactively. Applying a standardised algorithm for managing CD during and after the procedure is fundamental for optimising patient outcomes. This approach can help reduce the risk of PPMI and ensure that patients receive the most proper and prompt interventions.

In our study, despite further advancements in procedural and technical aspects of the G2 group, such as the EP and S3U, the incidence of PPMI has not shown a significant reduction compared to the G1 group. The PPMI remains a significant challenge in TAVR, even with newer-generation systems. Technically, TAVR, unlike surgical valve replacement, directly impacts the membranous septum—an area close to the main components of the conduction tree—thereby intensifying the risk of CD and subsequent PPMI requirements [17]. SE valves, composed of larger frames, apply more tension on the conduction tree than BE valves, explaining the constantly higher PPMI rates in SE compared to BE valves, even with newer models. As is well-known, RBBB, depth of valve implantation, and implantation mechanism (SE vs. BE) remain the strongest predictors of PPMI.

On the other hand, a significant finding from our analysis was the reduction in the incidence of new CD, especially LBBB, in G2, a technological advancement aimed at minimising cardiac electrical disruptions and demonstrating an enhanced safety profile for CD integrity.

The relationship between PPMI following TAVI and long-term mortality remains a subject of ongoing debate. Numerous studies, including a massive German Aortic Valve Registry analysis, have found a correlation between PPMI and increased mortality [26,27]. On the contrary, our review and other recent studies and meta-analyses indicate a lack of significant relation between PPMI and mortality. Moreover, at least one study suggests a protective role of PPMI against sudden cardiac death [28,29]. Although the prognosis after PPMI is still debatable, there is a general agreement on the link between PPMI and a higher risk of hospital admissions for heart failure post-TAVR [29,30]. Ventricular desynchrony caused by PPMI is the main cause of this adverse outcome [31,32]. Moreover, the impact of this association may depend on the pacing burden and could take over two years to become clinically evident. In summary, the relationship between PPMI and patient outcomes post-TAVR remains unclear and is debated, underscoring the need for further research to determine its independent effects on health outcomes.

Based on our results, G2 THV did not affect the one-month all-cause mortality rate. However, the one-year mortality rate is notably lower for patients treated with G2 than for those treated with G1. The slight technical differences between the valve generations alone do not account for these variations. Other factors, such as the inclusion of younger patients and those with moderate and low risk in TAVR procedures and the experience of the implanting physician, likely also play a significant role in these outcomes.

Our G2 THV aimed to simplify the TAVR procedure, as evidenced by statistically significant reductions in contrast agent use and procedural time compared with the G1 group. This reduction may indicate greater security among operators, potentially leading to fewer angiographies and reduced imaging. As this trend in TAVR procedures continues, it highlights a crucial area for further investigation. By studying and documenting these developments, we can further enhance the safety and efficiency of the procedures in the future.

Efforts to lower PPMI rates continue through pre-procedural adjustments and technical modifications [32]. Given that SE valves have been consistently linked with a significantly higher incidence of PPMI, various strategies have been used to reduce this occurrence. One of the modifications that is worth mentioning here is a new implantation technique called the cusps overlap view technique, which was invented using a coplanar projection that overlaps the right and left coronary cusps [33], which could allow for more accurate control over the depth of valve implantation, resulting in less contact with the septum conduction tree, resulting in less CD and subsequently less PPMI. Furthermore, MIDAS, another procedural approach (minimising depth according to the membranous septum), was also developed, significantly reducing PPMI risk by targeting a valve depth shorter than the membranous septum’s length, often achieving single-digit PPMI rates [34].

Several limitations of our study need attention. Firstly, our data collection was prospective, but the analysis was performed retrospectively. Retrospective analyses inherently carry a risk of bias. Secondly, the two study groups differed in size, with G2 approximately half the size of G1. We attempted to address this imbalance and reduce confounding by using IPTW based on a comprehensive propensity score model; however, IPTW cannot account for unmeasured confounders, and residual confounding cannot be excluded. By subcategorising the valve types, there are significant discrepancies in the number of implanted valves, especially the Acurate Neo and Acurate Neo 2, which were implanted only three and five times, respectively, resulting in 33% and 0% for both CD and PPMI rates, respectively. With a low implantation rate, we find the results are not reliable for evaluation. We mean that further investigation is needed to evaluate these THVs better. On the other hand, larger groups tend to be less affected by outliers and provide more robust data. Furthermore, a larger sample size often correlates with increased operator experience, potentially leading to lower complication rates and higher success rates.

## 5. Conclusions

The study shows a potential advantage of G2 THV in reducing post-procedure CD compared to G1, with no difference in the overall 30-day PPMI rate between the two groups. Subcategorisation based on manufacturing companies highlights varying PPMI incidences among G2 THV. The Sapien 3 Ultra valve significantly outperformed all other valve types and generations in reducing new CD and 30-day PPMI, emphasising its effectiveness. The deciding factors for CD and PPMI remain multifactorial, including patient selection, implantation technique and depth, valve release mechanism and PPMI risk factors. VARC III safety outcomes are still comparable between the G1 and G2 groups. These findings emphasise the need to carefully consider specific valves, patient selection and procedural characteristics to improve outcomes in TAVR, with ongoing research essential to refine consistency and long-term efficacy.

## Figures and Tables

**Table 1:** Valve generations.

Company	Older Generation (G1)	Newer Generation (G2)
Edwards Lifesciences (Irvine, CA, USA)	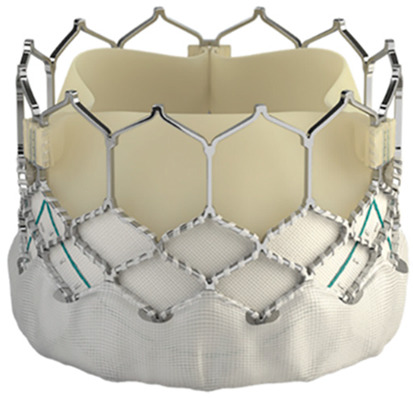	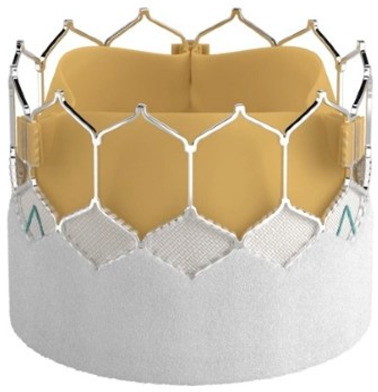
	Sapien 3 (Edwards Lifesciences) Mechanism: BEV New Characteristics: Notable for its transformational design with a sealing skirt to reduce PVL, optimised for ease of use and enhanced sealing. Previous Version: SAPIEN XT	Sapien 3 Ultra (Edwards Lifesciences) Mechanism: BEV New Characteristics: Enhanced outer skirt to prevent PVL, crucial for severe AS. New material for improved biocompatibility and sealing. Previous Version: SAPIEN 3
1-Edwards Lifesciences 2-Abbot (Chicago, IL, USA)	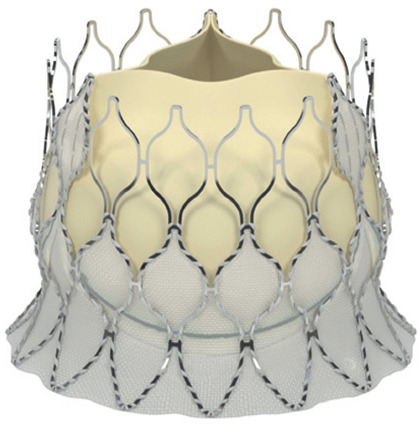	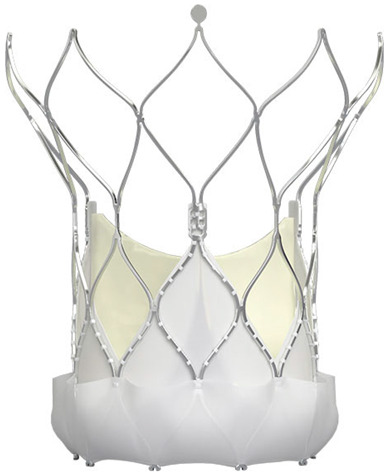
	1- Centera (Edwards Lifesciences) Mechanism: SEV New Characteristics: Motorised delivery system for simple, time-efficient procedures. Ultra-low profile for easy navigation. Previous version: new development	1- Navitor (Abbott) Mechanism: SEV New Characteristics: NaviSeal^TM^ Cuff for dynamic sealing, minimising leakage. Designed for immediate function and future coronary access. Previous Version: Portico System
Medtronic (Minneapolis, MN, USA)	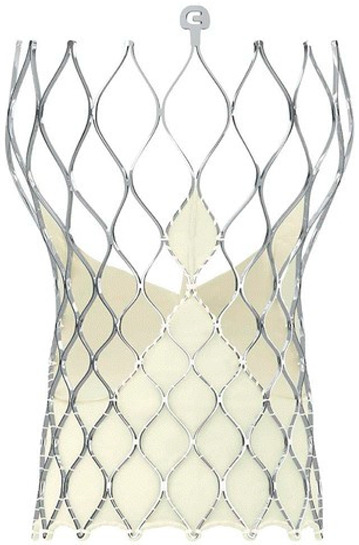	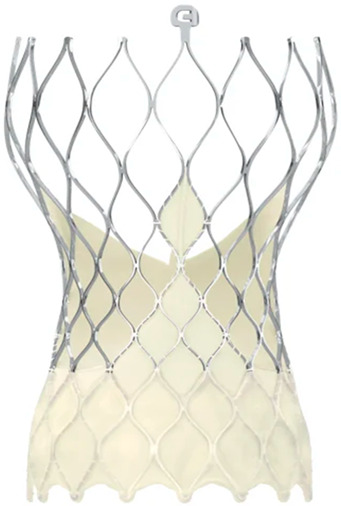
	CoreValve Evolut R (Medtronic) Mechanism: SEV New Characteristics: Conforming nitinol frame for better sealing. Recapture and repositioning capability for optimal deployment. Previous Version: CoreValve system	CoreValve Evolut PRO (Medtronic) Mechanism: SEV New Characteristics: External tissue wrap for increased sealing contact, advanced frame design to reduce PVL. Previous Version: Evolut R system
Boston Scientific (Marlborough, MA, USA)	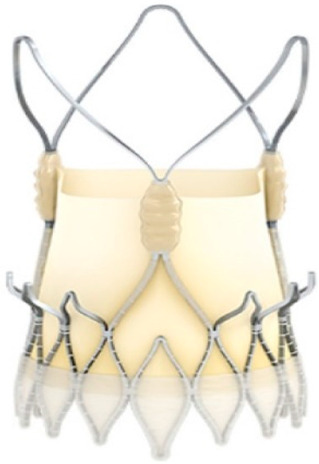 Acurate Neo (Boston Scientific) Mechanism: SEV New Characteristics: Designed to simplify the procedure with a stable deployment mechanism, aims to reduce PVL. Previous version: new development	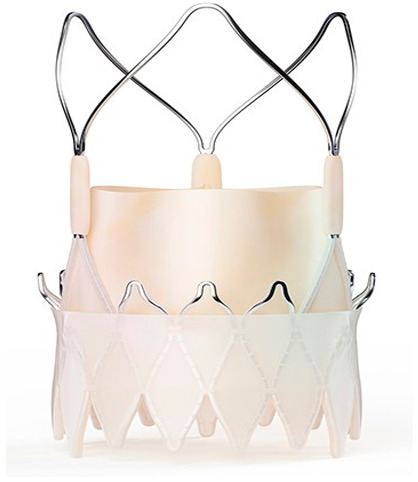 Acurate Neo2 (Boston Scientific) Mechanism: SEV New Characteristics: Active PVseal^TM^ technology for enhanced sealing. Precise placement system with dual anchoring. Open-frame design for future interventions. Previous Version: Acurate Neo

## Data Availability

The original contributions presented in this study are included in the article. Further inquiries can be directed to the corresponding author.

## Abbreviations

AS	Aortic Stenosis
AVB	Atrioventricular Block
BE	Balloon-Expandable
CD	Conduction Disturbance
EP	Evolut PRO
ER	Evolut R
G1	Older Generation of THV
G2	Newer Generation of THV
LBBB	Left Bundle Branch Block
LCC	Left Coronary Cusp
LVOT	Left Ventricle Outflow Tract
PPMI	Permanent Pacemaker Implantation
PVL	Paravalvular Leak
RBBB	Right Bundle Branch Block
S3	Sapien 3
S3U	Sapien 3 Ultra
SE	Self-Expanding
TAVR	Transcatheter Aortic Valve Replacement
THV	Transcatheter Heart Valves, Transcatheter Aortic Prostheses
